# Machine learning algorithms utilizing blood parameters enable early detection of immunethrombotic dysregulation in COVID‐19

**DOI:** 10.1002/ctm2.523

**Published:** 2021-09-26

**Authors:** Zhaoming Zhou, Xiang Zhou, Liming Cheng, Lei Wen, Taixue An, Heng Gao, Hongrong Deng, Qi Yan, Xinlu Zhang, Youjiang Li, Yixing Liao, Xin‐zu Chen, Bin Nie, Jie Cheng, Guanhua Deng, Shengqiang Wang, Juan Li, Hanqi Yin, Mengxian Zhang, Linbo Cai, Lei Zheng, Minglun Li, Bleddyn Jones, Longhua Chen, Amir Abdollahi, Meijuan Zhou, Ping‐Kun Zhou, Cheng Zhou

**Affiliations:** ^1^ Department of Radiation Oncology, Nanfang Hospital Southern Medical University Guangzhou China; ^2^ Department of Radiation Medicine, School of Public Health Southern Medical University Guangzhou China; ^3^ Department of Anesthesiology General Hospital of Central Theater Command of PLA Wuhan China; ^4^ Department of Laboratory Medicine, Tongji Hospital, Tongji Medical College Huazhong University of Science and Technology Wuhan China; ^5^ Department of Oncology Guangdong Sanjiu Brain Hospital Guangzhou China; ^6^ Department of Laboratory Medicine, Nanfang Hospital Southern Medical University Guangzhou China; ^7^ Department of Neurosurgery Jiangyin Affiliated Hospital of Southeast University School of Medicine Jiangyin China; ^8^ Department of Endocrinology and Metabolism, Guangdong Provincial Key Laboratory of Diabetology the Third Affiliated Hospital of Sun Yat‐sen University Guangzhou China; ^9^ Department of Geriatrics, Tongji Hospital, Tongji Medical College Huazhong University of Science and Technology Wuhan China; ^10^ Department of Cardiology, Nanfang Hospital Southern Medical University Guangzhou China; ^11^ Department of Clinical Laboratory, The Fourth Affiliated Hospital Zhejiang University School of Medicine Yiwu China; ^12^ Department of Critical Care Medicine, The First Affiliated Hospital Zhejiang University School of Medicine Hangzhou China; ^13^ Department of Gastrointestinal and Hernia Surgery, The Second People's Hospital of Yibin ‐ West China Yibin Hospital Sichuan University Yibin China; ^14^ Department of Gastrointestinal Surgery, West China Hospital Sichuan University Chengdu China; ^15^ Department of Laboratory Medicine, The Second People's Hospital of Yibin ‐ West China Yibin Hospital Sichuan University Yibin China; ^16^ Center for Reproductive Medicine, Renji Hospital School of Medicine Shanghai Jiao Tong University Shanghai China; ^17^ Shanghai Key Laboratory for Assisted Reproduction and Reproductive Genetics Shanghai China; ^18^ Department of Rehabilitation Medicine, Tongji Hospital, Tongji Medical College Huazhong University of Science and Technology Wuhan China; ^19^ South China Institute of Biomedicine Guangzhou China; ^20^ Department of Oncology, Tongji Hospital, Tongji Medical College Huazhong University of Science and Technology Wuhan China; ^21^ Department of Radiation Oncology University Hospital, Ludwig‐Maximilians‐University (LMU) Munich Munich Germany; ^22^ Gray Laboratory CRUK/MRC Oxford Institute for Radiation Oncology University of Oxford Oxford UK; ^23^ Translational Radiation Oncology German Cancer Research Center (DKFZ) and University Heidelberg School of Medicine Heidelberg Germany; ^24^ Department of Radiation Biology Beijing Key Laboratory for Radiobiology Beijing Institute of Radiation Medicine Beijing China

Dear Editor,

The pandemic of coronavirus disease 2019 (COVID‐19) has stressed and overloaded the existing medical capacity worldwide. From a more pragmatic perspective, the early detection of patients who may experience rapid clinical deterioration will enable prompt interventions and avert disease progression.^1^ T cell exhaustion, immunothrombotic dysregulation, as well as complement‐associated microvascular injury are considered as the hallmarks of disease severity in COVID‐19.^2^, ^3^, ^4^, ^5^ It is generally accepted that the identification of useful surrogates, for example, IL‐6, TNFα, MIP1α, LDH, ferritin, D‐dimer, CK, *etc*., to represent as immune response to COVID‐19 infection is crucial.^3^, ^4^, ^6^ Nevertheless, no individual parameter was so far predictive of immune‐thrombotic dysregulation fueled by a maladaptive host inflammatory response in severe infection with SARS‐CoV‐2.^7^, ^8^, ^9^ We, therefore, consider to develop potential solutions for forecasting thrombotic complications prior to clinicopathological exacerbation.

By incorporating whole blood transcriptome profiling and multi‐omics analysis, our study characterized immunological and hematological perturbations with respect to different categories of severity (*i.e*., healthy donors *vs*. mild or moderate *vs*. severe *vs*. critical illness). Functional diversity was found among those groups by unsupervised hierarchical clustering of differential expression profiles (Figure 1A, left). Circus plots revealed that the differentially expressed genes (DEGs) were enriched into the key processes, that is, neutrophil activation, platelet activation, blood coagulation, complement receptor‐mediated signaling pathway, leukocyte activation, and cytokines production. In contrast, the downregulated DEGs were functionally linked with lymphocyte activation/proliferation/differentiation/migration, gamma delta (γδ) and alpha beta (αβ) T cells activation, and so on (Figure 1A, right, and B). More specifically, the upregulation of gene‐signatures in platelet, neutrophil, and coagulation activation, as well as downregulation of lymphocyte activation in severe and critically ill COVID‐19 were demonstrated (Figure 1B).

**FIGURE 1:**
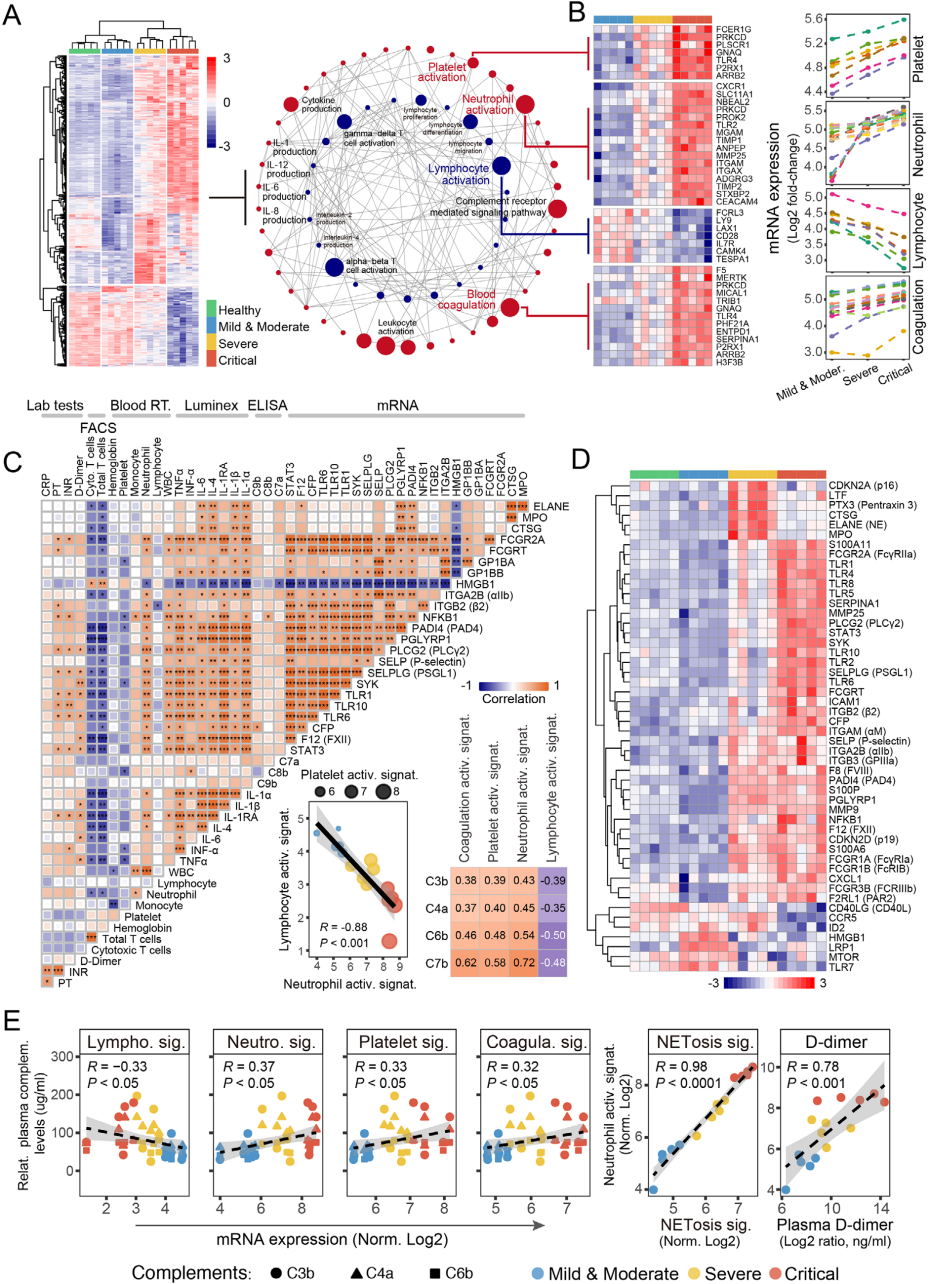
Transcriptional profiling of whole blood and multi‐omics characterization of peripheral immune response in COVID‐19 patients with different severity. (A) The diversity of gene‐expression profiles in patients with different severity shown in unsupervised hierarchical clustering (Left). A circus plot shows transcriptional regulatory pathways, as well as network profiles from Gene Ontology Biological Process (GO‐BPs) enrichment results of those differentially expressed genes (DEGs) (Right). The up‐ and down‐regulated GO‐BPs were represented by red and blue colors. (B) Heatmap and line charts display the differentially regulations of gene‐subsets for platelet, neutrophil, lymphocyte, and blood coagulation activations. (C) Multi‐omics characteristics correlation matrix of 43 features of COVID‐19 patients. Linear regression for the correlation of lymphocyte‐neutrophil‐platelet activity in COVID‐19. The square size corresponds to the absolute value of the Spearman rank correlation coefficient, with brown (blue) color indicating a positive (negative) correlation. *FDR < 0.05, **FDR < 0.01, ***FDR < 0.001. (D) Heatmap for gene‐signatures of activation, recruitment and interactions for neutrophil, platelet, and the formation of NETs (NETosis). (E) Correlation analysis for plasma complements or D‐dimer versus transcriptional levels of specific gene‐subsets

Multi‐omics data incorporating plasma cytokines and chemokines, circulating complements, flow cytometry‐derived immune cells counts, clinical laboratory outcomes, as well as featured gene‐signatures were implicated in pairwise Pearson correlations (Figure 1C, left). Furthermore, the upregulations of both neutrophil and platelet activation signatures were strongly correlated with downregulation of lymphocyte activation (*R *= –0.88, *p *< 0.001) (Figure 1C, middle). Gene‐subsets for neutrophil, platelet, and coagulation activations were found to correlate with blood complements C3b, C4a, C6b, and C7b, in contrast to lymphocytes as inverse correlations (Figure 1C, right, and 1E, left).

DEGs with specific interests to the recruitment and activation of neutrophils and platelets were also studied. A spectrum of genes were identified in initiation and amplification of the proinflammatory response, immune complex‐mediated activation of neutrophils, acting as cell surface receptors or their intracellular signal transductions for platelets and neutrophils, for example, S100As, SERPINA1, TLRs, STAT3, SELP (P‐selectin), SELPLG (PSGL‐1), SYK, F2RL1 (PAR2), ITGAM (αM), ITGB2 (β2), ITGA2B (αIIb), ITGB3 (GPIIIa), and so on. The key molecules associating with NET formation (NETosis), including PAD4, FCGR2A (FcγRIIa), PLCG2 (PLCγ2), CFP, F8, and F12, were considerably upregulated, facilitating platelets–neutrophils conjugates and highly procoagulant microcirculation disturbances via intrinsic pathways.^10^ Those transcriptional signatures were also partially evidenced in the proteomics level by Tian et al.^11^ Intriguingly, neutrophil effector molecules, such as ELANE (neutrophil elastase), MPO (myeloperoxidase), CTSG (Cathepsin G), as well as vascular inflammation mediator PTX3 and neutrophil‐derived lactoferrin, were significantly upregulated in severe compared to critical illness (Figure 1D). NETs were described as important mediators of coagulation.^12^ Neutrophil activations correlated well with NETosis (*R *= 0.98, *p *< 0.0001), as well as blood D‐dimer concentrations (*R *= 0.78, *p *< 0.001), highlighting a prominent role of activated neutrophils or NETosis in the pathogenesis of COVID‐19 coagulopathy (Figure 1E, right).

The unveiled transcriptional findings were validated in a multicenter cohort of 1219 eligible individuals (Figure S1). A summary of patient characteristics is provided (Table S1). Peripheral lymphocyte, neutrophil, platelet counts, as well as hemoglobin and ages among different severity groups were shown (Figure 2A–E). Besides, the demographically predictive of protection against advancement of severity in COVID‐19 is female sex, particularly for critically ill and lethal events (Figure S2). Consistent with transcriptional findings, clinical laboratory outcomes evidenced that lymphopenia, neutrophilia, as well as thrombocytopenia owning to the overconsumption of platelets were notably characterized in the late stages of COVID‐19. And those features were of mutual linkages and exhibited correlation to varying degrees (Figure 2F). A three‐dimensional simulation further implicated the dynamic interplay of lymphocyte, neutrophil, platelet, and hemoglobin (Figure 2G), providing a solid basis for mathematical modeling. Nonetheless, an individual blood parameter had relatively poor predictive performance for stratifying patients with different severity (Figure 2H–J and Table S2).

**FIGURE 2:**
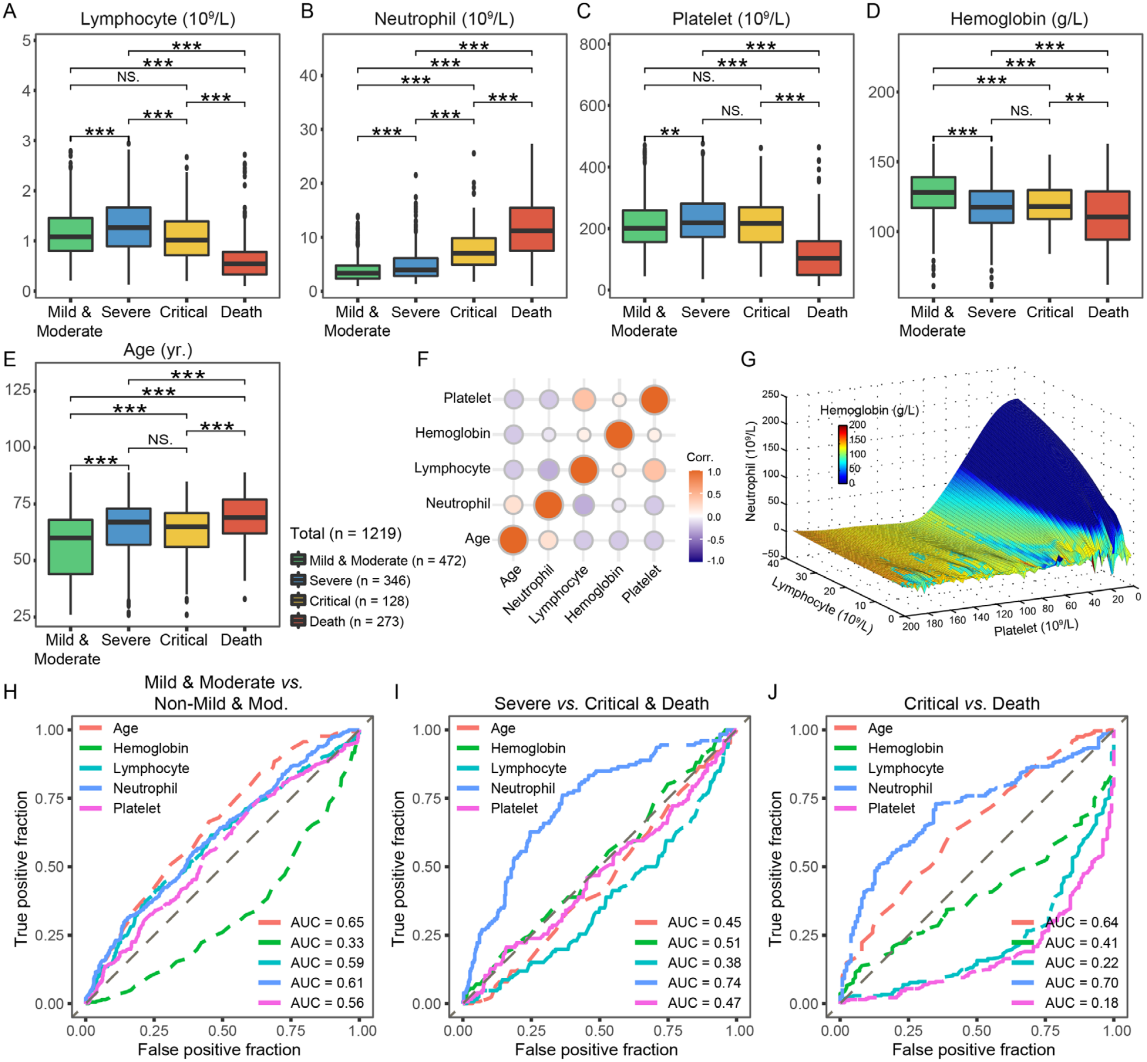
Critical blood parameters as well as age associated with disease severity of COVID‐19 patients. (A–E) Boxplots depicting lymphocyte, neutrophil, platelet, hemoglobin, and age with respect to disease severity. *p < 0.05, **p < 0.01, ***p < 0.001. (F) Correlation matrix of lymphocyte, neutrophil, platelet, and hemoglobin in the peripheral blood, as well as age in COVID‐19 patients. (G) Intuitive three‐dimensional plot shows the interplay of lymphocyte, neutrophil, platelet counts, and hemoglobin level in 1219 COVID‐19 patients. (H–J) The featured outcomes from routine blood tests and age were examined independently by ROC curves in discriminating disease severity of COVID‐19 patients

To improve the discrimination accuracy, machine learning‐based severity classification was performed. LASSO regression classifier was applied to train the model utilizing the featured blood‐parameters (Figure 3A). The calibration curve demonstrated a good consistence between the predicted and observed values and favorable predictive performance confirmed by receiver operating characteristic (ROC) analysis (Figure 3B–D). The discriminative ability was also assessed for testing and validation cohorts (Figure S3A–H). In parallel, the generalized linear model (GLM) and linear discriminant analysis (LDA) were utilized for the construction and optimization of disease discrimination. Strong discriminative capacities were achieved for both GLM (Figure 3E) and LDA (Figure 3F)‐based algorithms. Eventually, the overall cohort of 1219 patients was stratified into different degrees of severity with a robust hierarchical classification capacity (Figure 3G).

**FIGURE 3:**
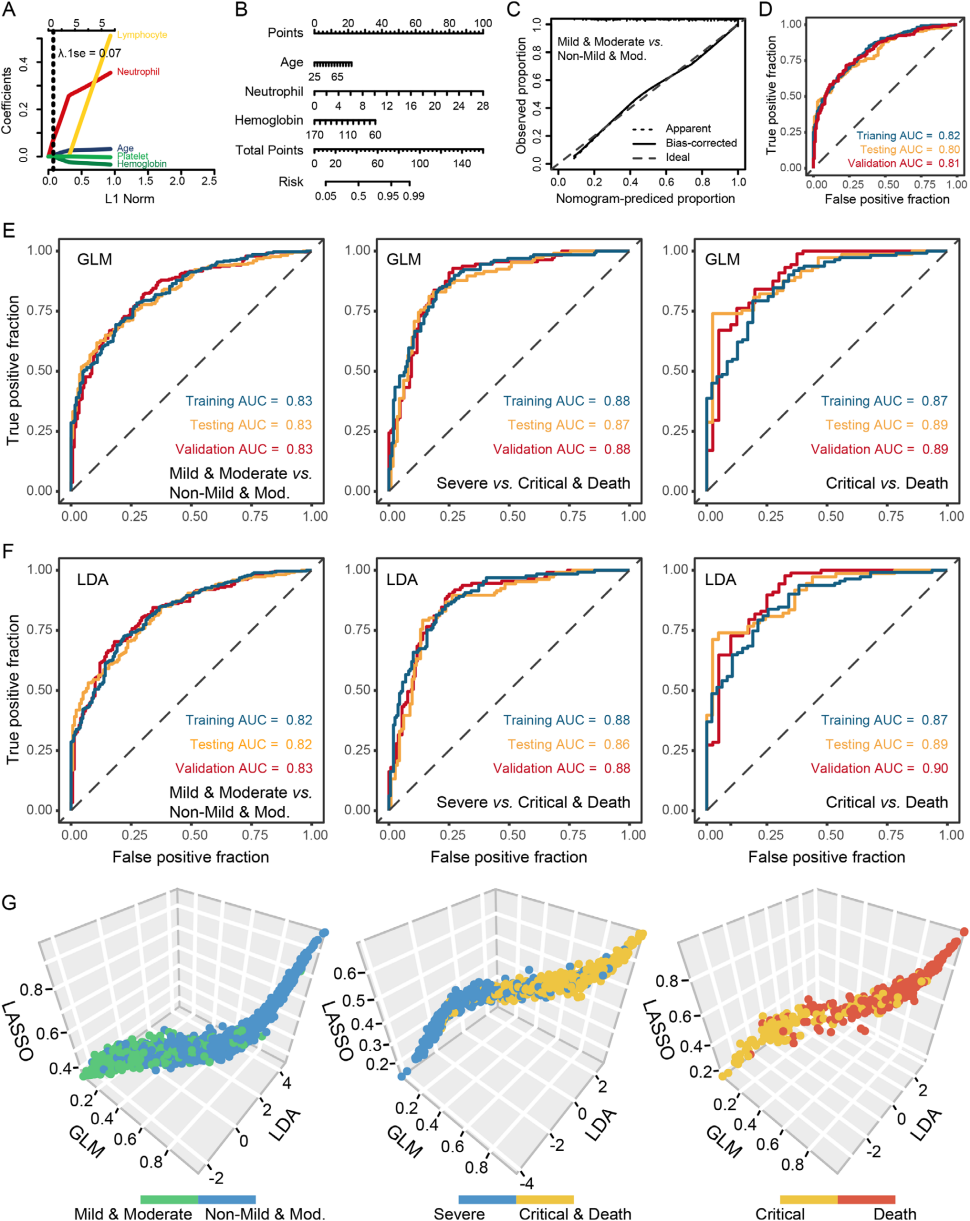
ROC curves depict classification accuracy of the machine learning models and the overview of the performance for machine learning‐based three‐dimensional severity classifications. (A–D) Construction, evaluation, and validation of LASSO‐based algorithms for disease severity classification. (E) GLM model. (F) LDA model. (G) Three‐dimensional classification plot shows the performance for machine learning‐based three‐dimensional severity classifications based on identified features

Machine learning‐based prognosis prediction was also studied (Figure 4A). The calibration curve and the diagonal coincided in general, indicating relatively high prediction accuracy for 15‐, 30‐, and 45‐days in‐hospital mortality risks (Figure 4B). A superior prediction capacity was demonstrated by decision curve analysis (DCA) and the net reduction in interventions was maximized (Figure 4C, D). The derived survival risk score was associated with immunethrombotic dysregulation. Patients in the training cohort could be, therefore, divided into high‐ and low‐risk groups with significantly stratified fatal risks (Figure 4E). Area under curves (AUCs) of 0.73 (95% CI, 0.65–0.81), 0.80 (95% CI, 0.73–0.87), and 0.81 (95% CI, 0.72–0.90) for 15‐, 30‐, and 45‐days were determined, and distinct survival outcomes were observed (*p *< 0.0001) (Figure 4F, G). Consistently, highly predictive performance was also evaluated in both internal testing (Figure 4H–J) and external validation cohort (Figure 4K–M).

**FIGURE 4:**
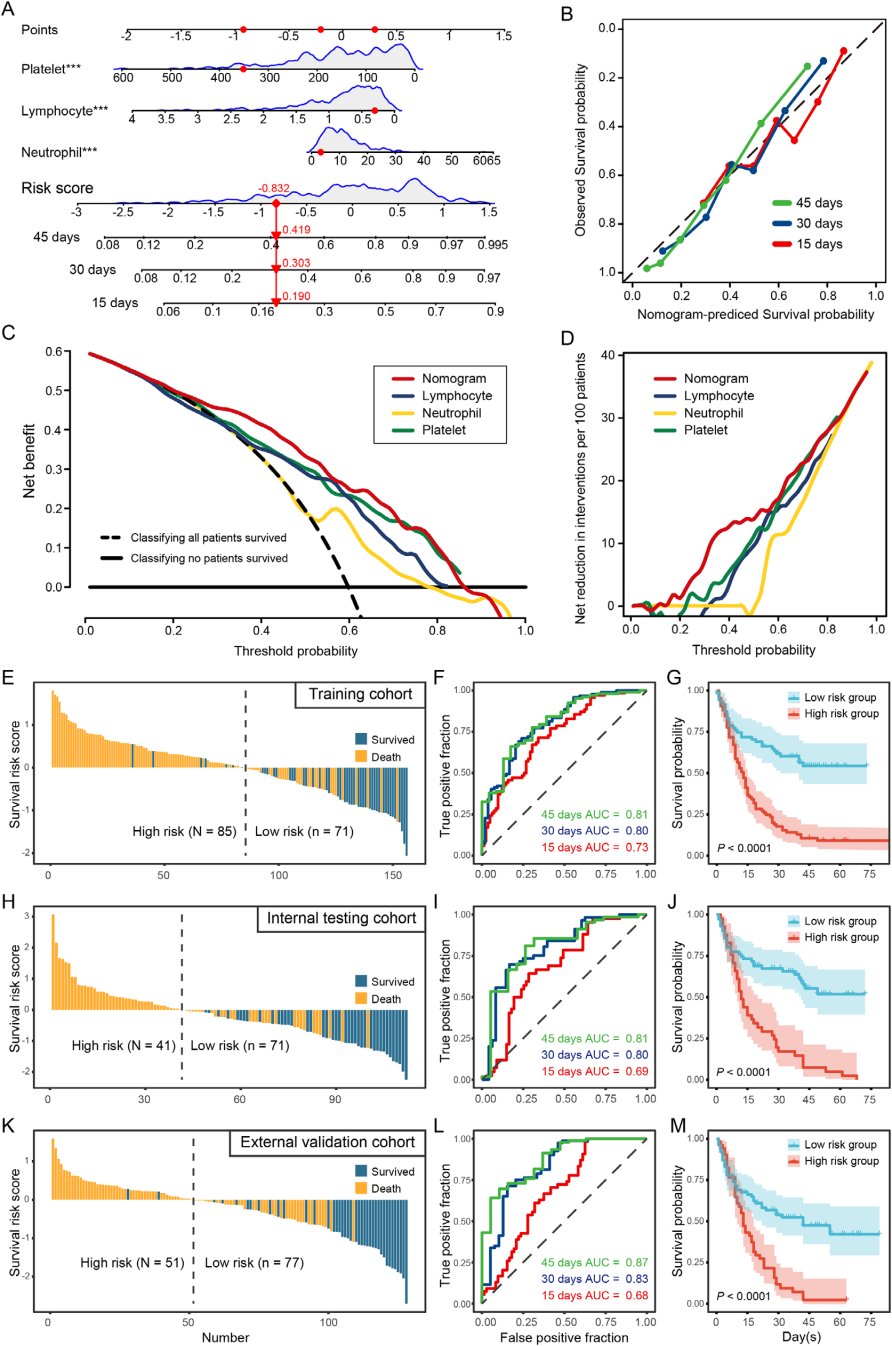
Construction, evaluation, and validation of algorithm for prognosis and fatal risks prediction. (A) LASSO Cox‐based nomogram in training cohort for predicting 15‐, 30‐, and 45‐days survival. Red dots represent a patient with total risk score is –0.832, overall death probabilities are 0.190, 0.303, and 0.419 within 15, 30, and 45 days, respectively. This patient is triaged as low risk. (B–D) Calibration, DCA, and net reduction plot for the nomogram. (E–G) The training cohort. (H–J) Internal testing cohort. (K–M) External validation cohort. We used AUCs at 15‐, 30‐, and 45‐days to assess prognostic accuracy, and calculated *p* values using the log‐rank test

In conclusion, genome‐wide whole blood profiling was performed to deciphering the peripheral immune and hematologic pertubations to COVID‐19, revealed an interesting feature of uncontrolled neutrophil‐complement‐coagulation interplay associated with immunethrombosis in severe and critically ill patients. Via machine learning techniques as well as the inclusion of large‐scale multicenter cohorts of 1219 patients, an optimized precision of prediction algorithm by integrating platelet, neutrophil, and lymphocyte counts and hemoglobin was established. Taken together, we developed and validated mechanistic‐driven rather than purely data‐driven algorithms to assess the specific risks of immunothrombotic dysregulation in COVID‐19. In principle, it might be used as a potential surrogate of decision‐making for the ICU patients with coagulation abnormalities, enabling more timely interventions, such as low molecular weight heparin‐treatment, and/or anticytokine therapies. Of note, those patients in ICUs are largely incapable of communicating and with very limited access to standard imaging utilizing computed tomography (CT). This algorithm will assist in guiding clinical decision‐making in more individualized managements and provide insights for longitudinal surveillance of severe and critically ill individuals.

## FUNDINGS

This work was supported by the National Natural Science Foundation of China (NSFC) (No. 81703166), Science and Technology Program of Guangzhou (Nos. 202002030445 and 202002030086), Natural Science Foundation of Guangdong Province (No. 2019A1515011943), China Postdoctoral Science Foundation (Nos. 2020T130052ZX and 2019M662974), and Medical Scientific Research Foundation of Guangdong Province (Nos. A2020505, A2020499, B2021203, and B2021139). The funders had no role in study design, data collection and analysis, decision to publish or preparation of the manuscript. All authors had full access to all the data in the study and had final responsibility for the decision to submit for publication.

## CONFLICTS OF INTEREST

The authors declare no potential conflicts of interest.

## ETHICS APPROVAL AND CONSENT TO PARTICIPATE

This study was approved by the Ethics Committee of Nanfang Hospital, Southern Medical University (approval number: NFEC‐2020‐033) and the Ethics Committees from the collaborated centers.

## AUTHOR CONTRIBUTIONS

CZ, ZZ, PZ, and LW conceived and designed the study. XZ, LMC, TA, HG, HD, QY, YJL, YXL, XC, BN, SW, XLZ, JL, MXZ, and HY assisted in acquisition, analysis, and interpretation of the data. ZZ, LW, CZ, DG, and CJ developed and validated the algorithms. ZZ, LW, DG, XZ, and LMC did the statistical and transcriptome analysis under the supervision of CZ, LHC, LBC, MLL, MJZ, and PZ. CZ, ZZ, and LW wrote the manuscript. BJ, AA, PZ, and LZ revised critically the study for important intellectual content. All authors have read and approved the final study.

## AVAILABILITY OF DATA AND MATERIALS

The transcriptome sequencing data was deposited at the Gene Expression Omnibus under the accession number GSE167930.

## Supporting information

Supporting information
