# Supplementary material for: Machine learning algorithms utilizing blood parameters enable early detection of immunethrombotic dysregulation in COVID‐19
Source: Clin Transl Med. 2021 Sep 26;11(9):e523. doi: 10.1002/ctm2.523 (PMC8473644; doi:10.1002/ctm2.523)
Supplement: Supplementary file 1 — Supporting information [file CTM2-11-e523-s001.docx]

**Supplementary appendix for**

**Machine Learning Algorithms Utilizing Blood Parameters Enable Early Detection of Immune-thrombotic Dysregulation in COVID-19**

**Authors:** Zhaoming Zhou^1 §^, Xiang Zhou^2 §^, Liming Cheng^3 §^, Lei Wen^4^, Taixue An^5^, Heng Gao^6^, Hongrong Deng^7^, Qi Yan^8^, Xinlu Zhang^9^, Youjiang Li^10^, Yixing Liao^11^, Xin-zu Chen^12,13^, Bin Nie^14^, Jie Cheng^15, 16^, Guanhua Deng^4^, Shengqiang Wang^17^, Juan Li^4^, Hanqi Yin^18^, Mengxian Zhang^19^, Linbo Cai^4^, Lei Zheng^5^, Minglun Li^20^, Bleddyn Jones^21^, Longhua Chen^22^, Amir Abdollahi^23^, Meijuan Zhou^1^, Ping-Kun Zhou^24^*, Cheng Zhou^22, 23^*

**Affiliations:**

^1^ Department of Radiation Medicine, School of Public Health, Southern Medical University, Guangzhou 510515, China

^2^ Department of Anesthesiology, General Hospital of Central Theater Command of PLA, Wuhan 430070, China

^3^ Department of Laboratory Medicine, Tongji Hospital, Tongji Medical College, Huazhong University of Science and Technology, Wuhan 430030, China

^4^ Department of Oncology, Guangdong Sanjiu Brain Hospital, Guangzhou 510510, China

^5^ Department of Laboratory Medicine, Nanfang Hospital, Southern Medical University, Guangzhou 510515, China

^6^ Department of Neurosurgery, Jiangyin Affiliated Hospital of Southeast University School of Medicine, Jiangyin 214400, China

^7^ Hongrong Deng, Department of Endocrinology and Metabolism, Guangdong Provincial Key Laboratory of Diabetology, the Third Affiliated Hospital of Sun Yat-sen University, Guangzhou 510120, China

^8^ Department of Geriatrics, Tongji Hospital, Tongji Medical College, Huazhong University of Science and Technology, Wuhan 430030, China

^9^ Department of Cardiology, Nanfang Hospital, Southern Medical University, Guangzhou 510515, China

^10^ Department of Clinical Laboratory, The Fourth Affiliated Hospital, Zhejiang University School of Medicine, Yiwu 322000, China

^11^ Department of Critical Care Medicine, The First Affiliated Hospital, Zhejiang University School of Medicine, Hangzhou 310003, China

^12^ Department of Gastrointestinal and Hernia Surgery, The Second People's Hospital of Yibin - West China Yibin Hospital, Sichuan University, Yibin 644000, China;

^13^ Department of Gastrointestinal Surgery, West China Hospital, Sichuan University, Chengdu 610041, China.

^14^ Department of Laboratory Medicine, The Second People's Hospital of Yibin - West China Yibin Hospital, Sichuan University, Yibin 644000, China

^15^ Center for Reproductive Medicine, Renji Hospital, School of Medicine, Shanghai Jiao Tong University, Shanghai 200135, China

^16^ Shanghai Key Laboratory for Assisted Reproduction and Reproductive Genetics, Shanghai 200135, China

^17^ Department of Rehabilitation Medicine, Tongji Hospital, Tongji Medical College, Huazhong University of Science and Technology, Wuhan, China, 430030

^18^ South China Institute of Biomedicine, Guangzhou 510535, China

^19^ Department of Oncology, Tongji Hospital, Tongji Medical College, Huazhong University of Science and Technology, Wuhan 430030, China

^20^ Department of Radiation Oncology, University Hospital, Ludwig-Maximilians-University (LMU) Munich, Munich D-81377, Germany

^21^ Gray Laboratory, CRUK/MRC Oxford Institute for Radiation Oncology, University of Oxford, Oxford OX3 7DQ, United Kingdom

^22^ Department of Radiation Oncology, Nanfang Hospital, Southern Medical University, Guangzhou 510515, China

^23^ Translational Radiation Oncology, German Cancer Research Center (DKFZ) and University Heidelberg School of Medicine, Heidelberg 69120, Germany

^24^ Department of Radiation Biology, Beijing Key Laboratory for Radiobiology, Beijing Institute of Radiation Medicine, Beijing 100850, China

^§^ Contributed equally to this article

*** Correspondence to:**

Cheng Zhou, M.D., PhD.

Department of Radiation Oncology

Nanfang Hospital, Southern Medical University

Guangzhou 510515, China

Email: czhou.rob@gmail.com

Phone: +86 20 6164 2136

Ping-Kun Zhou, M.D., PhD.

Department of Radiation Biology

Beijing Key Laboratory for Radiobiology, Beijing Institute of Radiation Medicine

Beijing 100850, China

Email: zhoupk@bmi.ac.can

Phone: +86 10 6693 1217

**Supplementary appendix**

**Table of Contents**

**Supplementary Methods ······················································································· 1**

**Supplementary Table 1 ·· ······················································································ 7**

**Supplementary Table 2 ························································································· 8**

**Supplementary Figure S1 ······················································································ 9**

**Supplementary Figure S2 ·····················································································10**

**Supplementary Figure S3 ···················································································· 11**

**Supplementary Methods**

**Study design and participants**

All patients diagnosed with COVID-19 were confirmed by a positive reverse-transcriptase polymerase chain reaction (RT-PCR) assay for SARS-CoV-2 RNA in a respiratory tract specimen. Patients were classified into different disease severity groups, *i.e.*, mild, moderate, severe, and critical illness according to the Guidance from World Health Organization as well as

National Institutes of Health^1,2^. To be specific, mild cases were defined as COVID-19 infected patients with no obvious symptoms and no radiological changes. Moderate cases were defined as fever or respiratory symptoms, with radiological evidences of pneumonia. Severe cases were defined as resting respiration ≥30 times/min or oxygen saturation ≤ 93% or Pa0_2_/Fi0_2_ < 300mmHg or lesions in chest imaging progressed > 50% within 24-48 hours. Critical cases were defined as respiratory failure occurs requiring mechanical ventilation or shock or organ failures requiring intensive care unit (ICU) treatment. Peripheral blood samples were derived from COVID-19 patients consistently to disease progression status, at the timing of the every disease severity confirmation, *i.e.*, mild, moderate, severe and critically illness. For example, blood draw was performed again once a moderate patient progressed into severe or critical illness upon re-assessment. For subjects who died, the results were included for analysis using the latest blood examinations during hospitalization. Clinical information and laboratory test outcomes of 1491 COVID-19 patients from 12 medical centers of different regions were collected, including routine blood tests, blood gas, blood chemistry, D-dimer, prothrombin time (PT), the international normalized ratio (INR), activated partial thromboplastin time (APTT), C-reactive protein (CRP), procalcitonin (PCT), lactate dehydrogenase (LDH), ferritin, *etc*. After exclusion of cases with incomplete clinical information or patients with primary hematologic diseases, a total of 1219 patients were included in this study. We used computer-generated random numbers to assign 470 of these patients to the training cohort, 360 patients to the internal testing cohort, and 389 patients to the external validation cohort. The clinical features were shown in supplementary materials (see ***Supplementary Table 1***). This study was approved by the Ethics Committee of Nanfang Hospital, Southern Medical University (*approval number: NFEC-2020-033*) and the Ethics Committees from the collaborated centers.

**Whole blood RNA-sequencing**

RNA-sequencing (RNAseq) was performed and analyzed as described ^3,4^. Briefly, after total-RNA isolation and DNase digestion probes were labeled and hybridized against Human Whole Genome BeadChip using illumina’s protocol ^5^ and sequenced the RNA on the Illumina Novaseq platform. With the processed transcriptome raw data, we selected the expressed genes and normalized them to counts per millions (CPM) for further analysis. Gene expression data was deposited at the Gene Expression Omnibus under the accession number GSE167930. The differential expression fold changes (FC) were represented by the log2 transformation. The differentially expressed genes (DEGs) were determined between different disease severity groups with the criteria that the adjusted *P* value < 0.05 and |log2FC| > 1.0. We calculated the adjusted *P* values using B-H correction. Hierarchical cluster analysis was performed on the DEGs among different groups utilizing method ‘correlation’ and DEGs clustered with ‘euclidean’ method. On the basis of the clustered DEGs, the Gene Ontology biological process (GO-BP) enrichment analysis was performed and the network profiles were displayed utilizing the ‘cytoscape’ software *v3.6.1*.

**Plasma cytokine and complement detection**

To determine multiple cytokines, chemokines and growth factors in plasma, Luminex assay with Cytokine/Chemokine/Growth Factor Convenience 45-Plex Human (*ThermoFisher*) was performed. The enzyme linked immunosorbent assay (ELISA) was applied for detecting and quantifying the levels of plasma complement components including C3a, C3b, C4a, C4b, C5a, C5b, C6a, C6b, C7a, C7b, C8a, C8b, C9a and C9b (*Human Complement ELISA Kits, Abcam*).

**Fluorescence activated cell sorter (FACS) detection**

To quantify the specific peripheral immune cells, flow cytometry was used for total T cells, cytotoxic T cells, monocytes, and NK cells counting. All samples were detected with the BD FACSCanto II flow cytometry system and analyzed with the BD FACSDiva software.

**Model development**

The baseline disease severity classification capability of each factor was assessed by single factor-based linear regression. To enhance the classification performance, we performed 3 regression-based machine learning (ML) methods, including least absolute shrinkage and selection operator (LASSO) regression method, linear discriminant analysis (LDA) method as well as generalized linear model (GLM) to build multi-factors-based classifier. When we have a single response variable Y with observations $y_{i}$, *i* = 1, ..., n, and a set of predictor variables $x_{i1}$,$x_{i2}$, ..., $x_{ip}$, the standard multiple linear regression model is:

$y_{i}= \alpha+ \sum_{j} \beta_{i}x_{ij}+ e_{i}$ (1)

where $e_{i}$ are residuals. COVID-19 disease severity status was considered as the event and performed a tenfold cross-validation in the training cohort to calculate the weight of LASSO penalty (denoted as lambda). The lambda with 1 SE of the minimum partial likelihood deviance was used for feature selection and the selected features were set as the input for the nomogram tool establishing and we tested the stability and accuracy of the models by calibration and ROC curves. We further performed the GLM and LDA methods to build the severity classification models based on the standard linear regression model using *equation (1)*. LASSO Cox regression method was used to select the prognosis associated features and build the prognosis classification nomogram. The nomogram provided the survival risk scores for each patient, which predicted the 15-day, 30-day, and 45-day mortalities and classified patients into high- and low- risk groups. The nomogram was assessed with the DCA, calibration, time-based ROC and survival curves.

**Algorithms for disease severity classification**

Clinical features were further selected and combined using several machine-learning based calculation methods, such as the least absolute shrinkage and selection operator (LASSO) regression method ^6^, the linear discriminant analysis (LDA) ^7^ method, and the generalized linear model (GLM) ^8^. Package ‘glmnet’ ^9^ was utilized for the LASSO method and package ‘MASS’ ^10^ was used to establish the LDA and GLM models. We used those ML methods to fit generalized linear models, by giving a symbolic description of the linear predictor and a description of the error distribution, with the purpose to identify which features were the most significant together with the level of significance as expressed by the corresponding coefficient of the projection hyperplane, as well as to classify unknown samples.

**Algorithm for prognosis prediction**

The LASSO Cox regression model was utilized to construct a multi-signature survival stratification nomogram in the training cohort. We performed a tenfold cross-validation on the training set to calculate the weight of LASSO penalty (denoted as lambda). The lambda with 1 SE of the minimum partial likelihood deviance was used for feature selection. Calibration curves were applied to evaluate the accuracy of the combined nomogram model. Decision curve analysis (DCA)^11^ was used to assess the clinical utility of the nomogram model established in the training cohort. Patients were divided into the high and low risk groups according to the risk scores computed by the nomogram in training cohort. The time-based ROC and Kaplan–Meier (KM) method was used for the survival analysis between the high risk and the low risk groups. The results were further validated in both the internal testing cohort and the external validation cohort.

**Statistics**

Pearson correlation analysis conducted using the multi-omics data including transcriptional signatures, plasma levels of complement, cytokines and chemokines, immune cells counts from flow cytometry, as well as laboratory outcomes. Nonparametric Kruskal-Wallis test was used for continuous variables comparisons between groups. The discrimination capacity was evaluated using the receiver operating characteristic (ROC) and the calculated the areas under the curves (AUCs) was used to test the accuracy of models. LASSO Cox regression model was used to do the multivariable survival analysis, and the Cox regression coefficients was used to generate nomograms. Kaplan-Meier curves were generated using the log-rank test to evaluate the differences between the survivals of different groups. Statistical analysis was performed using software *R version 3.5.1* and *P* < 0.05 was considered as statistically significant.

**Code and packages**

We performed the DEGs clustering with R package ‘pheatmap’. Samples were clustered with method ‘correlation’ and DEGs in all groups were clustered with ‘euclidean’ method. We calculated the pearson’s correlation confidences and *P* values were using R package ‘stats’ to observe the associations between clinical features. We generated the ROC curves and calculated the areas under the curves (AUCs) with R package ‘pROC’. We established the ML-based severity classification models with R package ‘glmnet’ and package ‘MASS’. We plotted the nomograms with R package ‘rms’. We used the R package ‘timeROC’ for the time-based ROCs generation. We generated the Kaplan-Meier curves and calculated the log-rank with R-packages ‘survival’ and ‘survminer’.

**References**

1. Reich DE, Cargill M, Bolk S, et al. Linkage disequilibrium in the human genome. *Nature* 2001; **411**(6834): 199-204.

2. LeBleu VS, Taduri G, O'Connell J, et al. Origin and function of myofibroblasts in kidney fibrosis. *Nature medicine* 2013; **19**(8): 1047-53.

3. Zhou C, Moustafa MR, Cao L, et al. Modeling and multiscale characterization of the quantitative imaging based fibrosis index reveals pathophysiological, transcriptome and proteomic correlates of lung fibrosis induced by fractionated irradiation. *Int J Cancer* 2019; **144**(12): 3160-73.

4. Cheng J, Zhou X, Feng W, et al. Risk stratification by long non-coding RNAs profiling in COVID-19 patients. *Journal of cellular and molecular medicine* 2021.

5. Parkhomchuk D, Borodina T, Amstislavskiy V, et al. Transcriptome analysis by strand-specific sequencing of complementary DNA. *Nucleic acids research* 2009; **37**(18): e123.

6. Tibshirani R. Regression shrinkage and selection via the lasso. *Journal of the Royal Statal Society, Series B* 1996; **58**(1).

7. Xanthopoulos P, Pardalos PM, Trafalis TB. Linear discriminant analysis. Robust data mining: Springer; 2013: 27-33.

8. Liang K-Y, Zeger SL. Longitudinal data analysis using generalized linear models. *Biometrika* 1986; **73**(1): 13-22.

9. Friedman J, Hastie T, Tibshirani R. glmnet: Lasso and elastic-net regularized generalized linear models. *R package version* 2009; **1**(4).

10. Ripley B, Venables B, Bates DM, et al. Package ‘mass’. *Cran R* 2013; **538**.

11. Fitzgerald M, Saville BR, Lewis RJ. Decision curve analysis. *Jama* 2015; **313**(4): 409-10.

| **Table S1. Clinical characteristics of 1219 patients with COVID-19** | | | | |
| --- | --- | --- | --- | --- |
|  | **Training cohort (N=470)** | **Testing cohort (N=360)** | ***P* value** | **Independent cohort (N=389)** |
| Gender |  |  |  |  |
| Male | 242 (52%) | 184 (51%) | 0.970 | 227 (58%) |
| Female | 228 (49%) | 176 (49%) |  | 162 (42%) |
| Age, years | 61.67 (20-95) | 63.73 (24-95) | 0.051 | 61.42 (10-96) |
| Severity |  |  |  |  |
| Mild/Moderate | 193 (41%) | 135 (38%) | 0.347 | 144 (37%) |
| Severe | 129 (27%) | 106 (29%) |  | 111 (29%) |
| Critical | 55 (12%) | 34 (9%) |  | 39 (10%) |
| Death | 93 (20%) | 85 (24%) |  | 95 (24%) |
| Event |  |  |  |  |
| Survived | 377 (81%) | 275 (76%) | 0.213 | 294 (76%) |
| Dead | 93 (20%) | 85 (24%) |  | 95 (24%) |
| Neutrophil, ×10^9^ per L | 6.35 (5.33) | 6.62 (5.57) | 0.468 | 6.78 (6.18) |
| Lymphocyte, ×10^9^ per L | 1.10 (0.63) | 1.11 (0.61) | 0.902 | 1.27 (2.46) |
| Hemoglobin, g/L | 119.51 (21.63) | 119.74 (20.01) | 0.877 | 121.32 (20.69) |
| Platelet, ×10^9^ per L | 201.09 (101.51) | 202.74 (106.24) | 0.820 | 196.66 (101.01) |
| Hospitalization, days | 24.88 (15.40) | 24.10 (13.90) | 0.453 | 24.17 (14.33) |
| Time in ICU, days | 4.50 (10.09) | 4.12 (8.36) | 0.573 | 5.16 (10.24) |

Data are n (%) or mean (SD).

| **Table S2. Clinical features for severity classification (ROC results)** | | | | | | |
| --- | --- | --- | --- | --- | --- | --- |
|  | Mild & Moderate *vs.*  Non-Mild & Mod. | | Severe *vs.*  Critical & Death | | Critical *vs.* Death | |
|  | AUC | 95% CI | AUC | 95% CI | AUC | 95% CI |
| Age | 0.65 | 0.61-0.69 | 0.45 | 0.4-0.51 | 0.64 | 0.58-0.70 |
| Neutrophil | 0.61 | 0.57-0.65 | 0.74 | 0.69-0.79 | 0.70 | 0.65-0.76 |
| Lymphocyte | 0.59 | 0.55-0.63 | 0.38 | 0.32-0.43 | 0.22 | 0.17-0.27 |
| Hemoglobin | 0.33 | 0.29-0.37 | 0.51 | 0.45-0.57 | 0.41 | 0.35-0.46 |
| Platelet | 0.56 | 0.52-0.6 | 0.47 | 0.41-0.53 | 0.18 | 0.14-0.22 |

**Figure S1. Flowchart of study participants for clinical validations and datasets for algorithm development.**


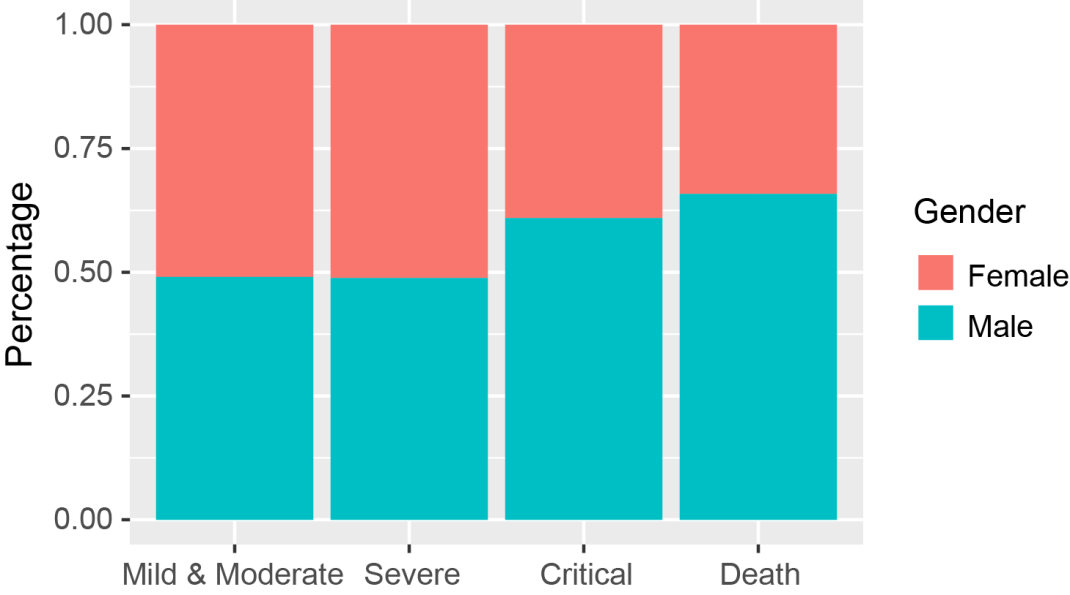


**Figure S2. The distribution of female and male sex in different degrees of severity of COVID-19 patients in our multi-center cohort of 1219 eligible patients.** The percentage of female sex is likely the same with males in mild & moderate as well as severe group. The distribution of female sex is relatively low in critical ill and death group compared to the males.

**Figure S3. Construction, evaluation and validation of LASSO-based algorithms for disease severity classification.** **(A, E)** Construction of the classifier. LASSO coefficient profiles of patient age, lymphocyte, neutrophil, platelet counts, and hemoglobin level. A vertical line is drawn at the value chosen by 10-fold cross-validation. **(B, F)** Nomograms to predict probabilities of disease severity. **(C, G)** Calibration curves suggest a good consistence between the prediction and observation. **(D, H)** ROC curves depict classification accuracy of the models in training, testing, and validation cohorts.
